# Impact of intravenous fluid administration on cardiac output and oxygenation during cardiopulmonary resuscitation

**DOI:** 10.1186/s40635-023-00497-4

**Published:** 2023-03-24

**Authors:** Jennifer Lutz, Yosef Levenbrown, Md Jobayer Hossain, Anne Hesek, Kelly E. Massa, James P. Keith, Thomas H. Shaffer

**Affiliations:** 1 Division of Pediatric Critical Care, Nemours Children’s Health, 1600 Rockland Road, Wilmington, DE 19803 USA; 2 Department of Pediatrics, Sidney Kimmel Medical School of Thomas Jefferson University, Philadelphia, PA USA; 3 Nemours Biomedical Research, Nemours Children’s Health, Wilmington, DE USA; 4 Department of Applied Economics and Statistics, University of Delaware, Newark, DE USA; 5 Department of Respiratory Care, Nemours Children’s Health, Wilmington, DE USA; 6 Nemours Biomedical Research/Research Lung Center, Nemours Children’s Health, Wilmington, DE USA; 7 Department of Pediatrics, Lewis Katz School of Medicine at Temple University, Philadelphia, PA USA

**Keywords:** Advanced cardiac life support, Basic cardiac life support, Cardiac arrest, Out-of-hospital cardiac arrest, Cardiac output

## Abstract

**Background:**

The effect of intravenous fluid (IVF) administration during cardiopulmonary resuscitation (CPR) is an unexplored factor that may improve cardiac output (CO) during CPR. The aim of this study was to determine the effect of IVF administration on CO and oxygenation during CPR.

**Methods:**

This experimental animal study was performed in a critical care animal laboratory. Twenty-two Landrace-Yorkshire female piglets weighing 27–37 kg were anesthetized, intubated, and placed on positive pressure ventilation. Irreversible cardiac arrest was induced with bupivacaine. CPR was performed with a LUCAS 3 mechanical compression device. Pigs were randomized into IVF or no-IVF groups. Pigs in the IVF group were given 20 mL/kg of Plasma-Lyte (Baxter International, Deerfield, IL USA), infused from 15 to 35 min of CPR. CPR was maintained for 50 min with serial measurements of CO obtained using ultrasound dilution technology and partial pressure of oxygen (PaO_2_).

**Results:**

A mixed-effects repeated measures analysis of variance was used to compare within-group, and between-group mean changes in CO and PaO_2_ over time. CO and PaO_2_ for the piglets were measured at 10-min intervals during the 50 min of CPR. CO was greater in the IVF compared with the control group at all time points during and after the infusion of the IVF. Mean PaO_2_ decreased with time; however, at no time was there a significant difference in PaO_2_ between the IVF and control groups.

**Conclusions:**

Administration of IVF during CPR resulted in a significant increase in CO during CPR both during and after the IVF infusion. There was no statistically significant decrease in PaO_2_ between the IVF and control groups.

## Background

Cardiopulmonary resuscitation (CPR) is the cornerstone of resuscitation in cardiac arrest. However, it is imperfect, with a survival rate of less than 15% [1]. Cardiopulmonary resuscitation generates 15 to 20% of native cardiac output (CO) [1]. In addition, perfusion and oxygen delivery to the organs during cardiac arrest is further limited by the body undergoing a systemic inflammatory response with a loss of vascular tone. As the duration of CPR progresses, this can result in loss of central blood volume and a drop in CO [2].

Globally, out-of-hospital cardiac arrest (OHCA) ranges from 20 to 40 per 100,000 people, with a survival rate of only 2–11%. In the United States alone, half a million people, including children, have a cardiac arrest, and less than 10% survive, making cardiac arrest the most lethal public health problem in the United States. Survival rates from cardiac arrest have not improved in many years, as methods to promote greater blood flow have been elusive [3]. Some providers administer intravenous fluids (IVF) during CPR to increase circulating blood volume and improve CO. However, variability in practice regarding the use of IVF during CPR persists, as there is a paucity of evidence regarding its use [4]. Pediatric OHCA is less common than in adults; however, the incidence of pediatric cardiac arrest in the United States is still 8.3 per 100,000 people, with just under 10% of these children surviving to hospital discharge [1]. In addition, the CO during CPR is less than 20% of its native CO at best, resulting in severely diminished oxygen delivery to the organs [1]. The current Advanced Cardiac Life Support and Pediatric Advanced Life Support Guidelines do not make recommendations regarding the use of IVF during CPR [5–7]. Even if the administration of IVF during CPR does augment CO, many providers are hesitant to administer IVF, with a concern that fluids administered during CPR can result in pulmonary edema secondary to the poor CO seen with CPR, which may hamper oxygen delivery during CPR. With OHCA survival being less than 10%, it is critical to assess what modifiable factors contribute to this dismal outcome. One area that remains unclear is the effect of IVF administration during CPR [3].

The primary aim of this study was to determine whether the administration of IVF has a favorable impact on CO and oxygenation during CPR using a porcine model of cardiac arrest. There have been no prior studies that have directly measured the effect of IVF on CO and arterial oxygenation during CPR.

## Methods

### Anesthesia and monitoring

This study was performed with 22 Landrace-Yorkshire juvenile pigs weighing 27–37 kg. The animals were purchased from the same supplier and provided to our laboratory on the day of the experiment and were not housed within our facility prior to the experiment. The Nemours Institutional Animal Care and Use Committee approved the experimental protocol. The care and handling of the animals were in accordance with National Institutes of Health guidelines. Female (due to availability) Landrace-Yorkshire juvenile pigs were chosen because their cardiovascular physiology is similar to humans making for a good CPR model. The body habitus is also accommodating for the LUCAS 3 mechanical compression device (Stryker, Kalamazoo, MI, USA). The animals underwent full health assessment and certification before transfer to our laboratory. Allocation of the animals to the IVF group or the control group was via a lottery system, using a paper lottery.

Each pig received initial sedation with two intramuscular injections of 1 mL/kg of an anesthetic cocktail composed of ketamine 23 mg/mL, acepromazine 0.58 mg/mL, and xylazine 0.8 mg/mL, administered 10 min apart. The pigs were then given a 5 mL intravenous injection of ketamine 10 mg/mL and propofol 10 mg/mL. Additional doses were administered as needed for signs of discomfort during the procedures. Dosing these medications was based on a protocol used in our laboratory in previous studies [8]. After adequately sedating the pigs, they underwent tracheal intubation via tracheostomy, using a 7.0–7.5 cuffed endotracheal tube. They were connected to a mechanical ventilator (Servo-I, Getinge, Wayne, NJ USA) and ventilated using a volume control mode of ventilation. The ventilator was set to a tidal volume of 8 mL/kg, initial respiratory rate of 20 breaths per minute, positive end-expiratory pressure (PEEP) 5 cmH_2_O, and the fraction of inspired oxygen (FiO_2_) 1.0, with subsequent adjustments to respiratory rate to maintain pH at 7.35–7.45. A PEEP of 5 cmH_2_O was maintained throughout the study, as this level of PEEP has been shown in prior studies in our laboratory to provide optimal CO and oxygenation during CPR [8]. Following intubation, an intra-arterial catheter was placed into the left carotid artery, and a central venous catheter was placed into the right internal jugular vein. Continuous infusions of propofol 7 mg/kg/h and ketamine 15 mg/kg/h were initiated. These infusions were increased if the pig demonstrated signs of pain (such as flinching or an increase in heart rate or blood pressure of 10% or greater to a painful stimulus). Sedation was also increased if needed for ventilator desynchrony. Dosing of the intravenous anesthetics was based upon recommendations from previous studies [8, 9]. After sedation was deemed adequate, pigs were given a 30-min stabilization period with ongoing monitoring of vital signs, including heart rate, blood pressure, respiratory rate, and end-tidal carbon dioxide.

### Experimental protocol

The COstatus CO monitor (Transonic Systems, Ithaca, NY USA) was used to measure CO via ultrasound dilution technology and has been validated for measurement of CO in both human and pig subjects when compared with the gold standard of thermodilution [10–14]. Baseline measurements of CO, blood pressure, and heart rate were obtained. In addition, baseline arterial blood gas measurements were obtained before cardiac arrest was induced using an iStat1 analyzer (Abbott Medical, Abbott Park, IL, USA). Nine mg/kg of bupivacaine was administered intravenously to induce cardiac arrest. This has been shown to cause irreversible cardiac arrest in previous porcine studies [2, 15]. Cardiac arrest was confirmed by asystole on the cardiac monitor, loss of end-tidal carbon dioxide reading, loss of pulse oximetry measurement, and loss of a pulsatile waveform on the arterial line tracing. When these were noted, the pig was left for 1 min to ensure that it did not return to a perfusing rhythm. After 1 min of cardiac arrest, a LUCAS 3 mechanical compression device (Stryker) was placed on the pig to provide compressions at a rate of 102. The performance of high-quality CPR was monitored by ensuring that end-tidal carbon dioxide was over 10 mmHg at all times.

The pigs assigned to the IVF group received a 20 mL/kg infusion of isotonic fluid with Plasma-Lyte (Baxter International, Inc., Deerfield, IL, USA). The IVF infusion began 15 min after chest compressions were initiated and infused over 20 min. The 15-min delay until the initiation of IVF was meant to simulate actual life circumstances. If fluid is given during CPR, the fluid administration would likely be delayed for a few minutes following the cardiac arrest and initiation of CPR. Plasma-Lyte was chosen, given its relatively neutral effect on pH compared with 0.9% normal saline. Cardiac output and arterial blood gases were measured every 10 min following the initiation of chest compressions for a total of 50 min for both the IVF and the control groups.

This study was powered for the primary outcome, which is the effect of IVF on CO during CPR. Assuming normal CO of 2.3 L/min for pigs this age [16] and assuming CPR yields 20% of normal CO resulting in an expected CO of 0.46 L/min ± 0.2 during CPR using an alpha of 0.05, a power of 0.8, and looking for a greater than 50% increase in CO in the group that received IVF compared with the group that did not receive IVF (which would increase the CO from 0.46 to 0.7 L/min), we calculated that a total of 22 subjects were needed for this study.

**Fig. 1:**
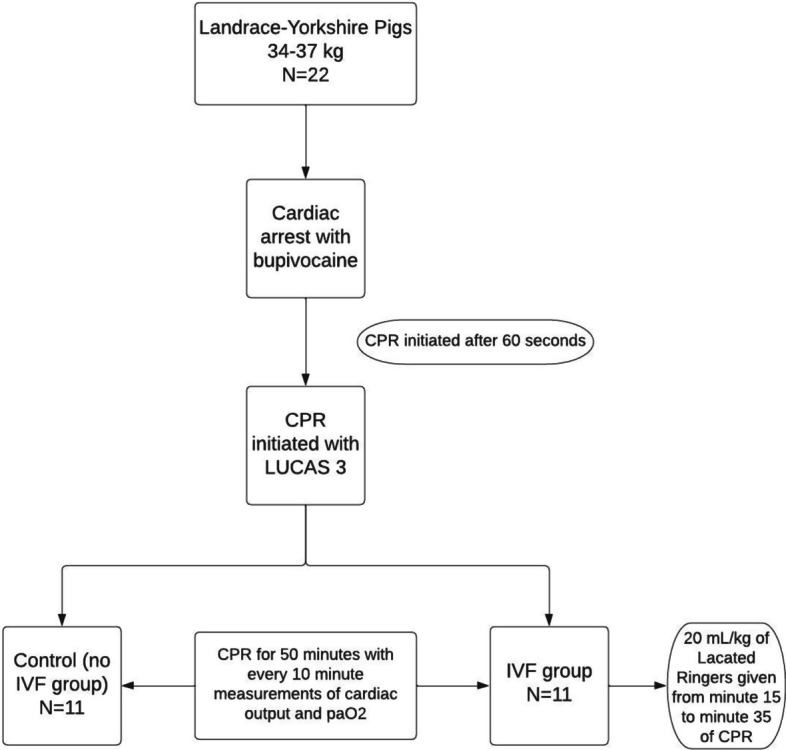
Flow diagram demonstrating summary of the experimental protocol

The experimental protocol is summarized in Fig. 1.

### Statistical analysis

Baseline characteristics, including CO, partial pressure of oxygen (PaO_2_), systolic blood pressure, diastolic blood pressure, and heart rate, were summarized using mean and standard error of mean by study groups. A two-sample t-test was used to compare the mean baseline characteristics of piglets between the two groups. A mixed-effects repeated measures analysis of variance was used to compare within-group, and between-group mean changes in CO and PaO_2_ over time after adjustment for the baseline values. Cardiac output and PaO_2_ were used as the response variables; piglets were used as the random effect; and intervention group, measurement time points, and corresponding baseline values were used as fixed effects in each model. The least significant difference test was used to conduct pairwise comparisons of least squared means between two study groups at different time points and within-group two-time points. Model assumptions were checked before analysis. All tests were two-tailed at an overall level of significance of 0.05. Statistical software SAS version 9.4 (Cary, NC, USA) was used for the analysis.

## Results

As shown in Table 1, summarized baseline mean blood pressure and heart rate were similar between the IVF group (*n* = 11) and the control group (*n* = 11). Table 2 demonstrates no significant difference in height and weight between the two groups (with the height measured from outstretched snout to outstretched foot). There was a difference in baseline CO, with the mean CO in the IVF group being 2.57 L/min and the mean CO in the control group 3.48 L/min (*p*-value = 0.04). This difference persisted at the 10-min measurement. However, because IVF was not started until 15 min into the study, this difference at 10 min reflects the baseline difference between the two groups.

**Table 1:** Between-group comparison of physiologic parameters at baseline and at 10 min

Variables	Time (min)	Control Mean (SEM)	IVF Mean (SEM)	*P*-value
Cardiac output (L/min)	Baseline	3.48 (0.35)	2.57 (0.20)	0.04
Partial pressure of oxygen (mmHg)	Baseline	422.80 (35.93)	460.90 (18.23)	0.36
Cardiac output (L/min)	10 min post-arrest	1.01 (0.12)	0.68 (0.08)	0.03
Partial pressure of oxygen (mmHg)	10 min post-arrest	167.90 (43.95)	159.60 (30.56)	0.88
Systolic blood pressure (mmHg)	Baseline	81.45 (2.83)	79.64 (3.34)	0.68
Diastolic blood pressure (mmHg)	Baseline	54.45 (3.41)	52.82 (2.95)	0.66
Heart rate (beats/min)	Baseline	93.64 (7.30)	82.36 (5.31)	0.23

*IVF* intravenous fluid, *SEM* standard error of mean

**Table 2:** Baseline height and weight of control and IVF groups

Variable	Control	IVF	*P*-value
*IVF height*			
Mean (SEM)	120 (1.6)	120 (1.2)	0.8971
Median (min, max)	120 (120, 130)	120 (120, 130)	
*IVF weight*			
Mean (SEM)	36 (0.23)	36 (0.27)	0.8003
Median (min, max)	36 (34, 37)	36 (34, 37)	

*IVF*  intravenous fluid, *SEM*  standard error of mean

**Fig. 2:**
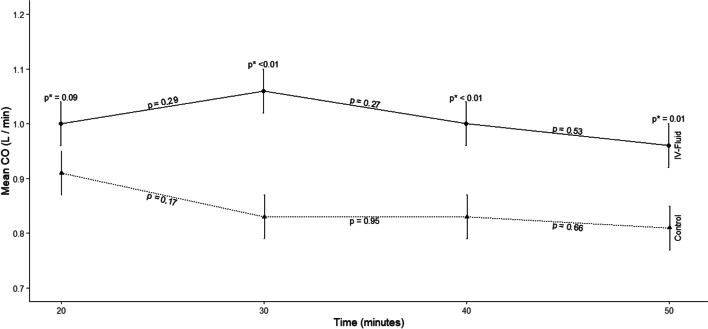
Mean values ± standard error of cardiac output for control and IVF groups as a function of time. As shown, *p* values demonstrate differences as a function of group and time after adjustment for baseline differences

Figure 2 and Table 3 both demonstrate the effect of IVF on CO during CPR after adjusting for baseline (including the 10 min) difference in CO between the two groups. As seen in Fig. 2, once the IVF was started (at 15 min post-cardiac arrest), CO was greater in the IVF group compared with the control group at all time points. In addition, the pairwise differences in CO between the IVF group and the control group at 30, 40, and 50 min were statistically significant.

**Table 3:** Between-group comparison of cardiac output and partial pressure of oxygen following initiation of intravenous fluid infusion

Variable	Time (min)	Control Mean (SEM)	IVF Mean (SEM)	*P*-value
Cardiac output (L/min)	20.00	0.91 (0.04)	1.00 (0.04)	0.0899
Cardiac output (L/min)	30.00	0.83 (0.04)	1.06 (0.04)	0.0001
Cardiac output (L/min)	40.00	0.83 (0.04)	1.00 (0.04)	0.0030
Cardiac output (L/min)	50.00	0.81 (0.04)	0.96 (0.04)	0.0051
Partial pressure of oxygen (mmHg)	20.00	151.09 (14.48)	144.55 (14.48)	0.7502
Partial pressure of oxygen (mmHg)	30.00	131.54 (14.48)	128.27 (14.48)	0.8735
Partial pressure of oxygen (mmHg)	40.00	112.54 (14.48)	105.82 (14.48)	0.7435
Partial pressure of oxygen (mmHg)	50.00	107.64 (14.48)	98.73 (14.48)	0.6649

*IVF*  intravenous fluid, *SEM*  standard error of mean

**Fig. 3:**
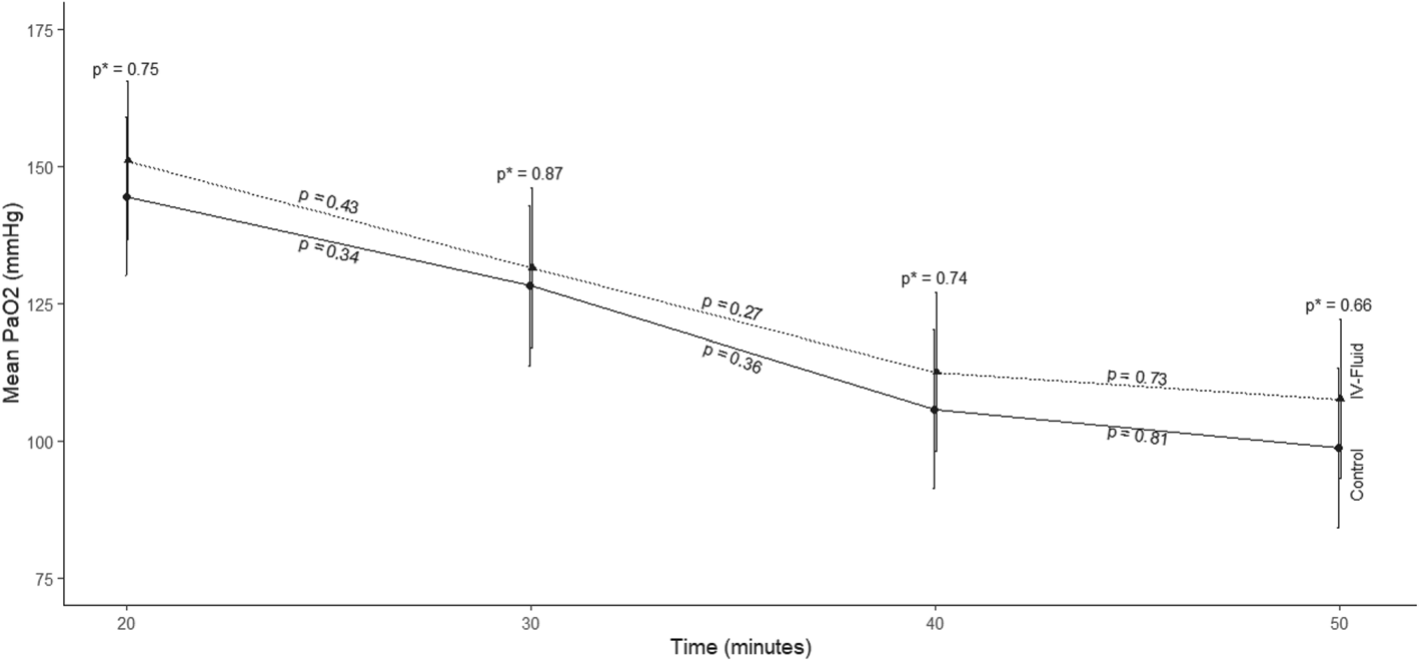
Mean values ± standard error of partial pressure of oxygen for control and IVF groups as a function of time. As shown, *p* values demonstrate differences as a function of group and time after adjustment for baseline differences

Figure 3 and Table 3 demonstrate the summarized effects of IVF on PaO_2_ during CPR. As shown in the figure, although the PaO_2_ decreased with time, at no time point was there a significant difference in PaO_2_ between the IVF group and the control group. Additionally, at all time points, the PaO_2_ was above 100 mmHg in both groups, except for the measurement at 50 min in the IVF group, where the PaO_2_ was 98.7 mmHg.

Adverse events included data from one animal that was disqualified due to the development of a massive pericardial effusion during CPR. In addition, data from four other animals were disqualified due to equipment malfunction during the study. Therefore, data from these animals are not included in the results of this study.

## Discussion

The aim of this study was to determine the effect of IVF administration during CPR on CO and PaO_2_ in the blood as a marker of oxygen delivery. The hypothesis of this study group was that administration of IVF during CPR will augment CO. The results of this study demonstrated that, from the time the IVF administration was started, there was an increase in CO at all time points in the group that received IVF during CPR compared with the control group. This difference was significant at 15, 25, and 35 min after initiating the IVF infusion. Although there was a decline in PaO_2_ with time during CPR, at none of the time points was there a significant difference in PaO_2_ between the IVF group and the control group. Changes in PaO_2_ between the two groups at a magnitude this study was powered to detect were likely not present between the two groups.

The increase in CO attributed in this study to IVF administration during CPR is likely due to the fluid's effect on the mean systemic filling pressure and subsequently on venous return. Cardiac output requires adequate filling of the ventricles, which during CPR occurs during the relaxation phase, followed by compression of the ventricles to expel the blood out of the ventricles. Typically, under physiologic conditions, the driving force for blood to return to the heart (preload) is the pressure difference between the mean systemic filling pressure (MSFP), which is the pressure that the blood exerts on the large veins in a static, no-flow state, and the right atrial pressure (Pra) [8, 17, 18]. In addition, the elastic recoil of the venous system contributes to the forward flow of blood [15]. Venous return, and secondarily CO, can be augmented by increasing the stressed volume of venous blood, which is the volume of blood that puts pressure on and stretches the walls of the venous system, which contributes to the MSFP. It has been postulated that in a state of low sympathetic tone, such as what could be expected in a cardiac arrest patient, only 25 to 30% of the intravascular volume will constitute the stressed volume [15]. The stressed volume of blood can be increased either by increasing the total blood volume or decreasing the diameter of the vessels in the venous system [19–22].

In a series of classic experiments looking at cardiovascular physiology, Guyton and colleagues demonstrated that lowering the right atrial pressure produces a linear increase in venous return [15, 21, 23, 24]. However, venous return is limited when the pressure in the great veins is lower than the pressure outside their walls due to the collapse of the vessels under those conditions [2, 15]. When venous collapse occurs, further lowering of the right atrial pressure does not increase venous return [25]. Thus, the best the heart can do to augment venous return is to reduce the Pra to zero, at which point further lowering of the Pra will not lead to increased venous return due to the collapse of the large veins. Because vessel collapse and flow limitations occur at atmospheric and even at supra-atmospheric pressures [26] in patients ventilated with positive pressure ventilation, there is a significant risk that venous return and CO will be limited during CPR secondary to vessel collapse. When the venous return is limited this way, CO can only be augmented by increasing the MSFP, which can be done most effectively by a volume infusion during CPR (although MSFP can also be increased by decreasing vessel diameter, this may be hard to accomplish during a cardiac arrest if there is a loss of vasomotor tone). By demonstrating an increase in CO during CPR with IVF administration, this study demonstrates that increasing the stressed blood volume can augment CO during CPR, as postulated above. Thus, this study confirms that these basic principles of cardiovascular physiology will hold true in cardiac arrest during CPR.

There are also potential downsides of IVF administration during CPR. Since the CO during CPR is approximately 15 to 20% of native CO, it is possible that administering additional fluids can elevate the right atrial pressure, further impeding right ventricular filling and thereby decreasing CO. However, this outcome was not encountered in our study. In addition, IVF administration during CPR can lead to the development of cardiogenic pulmonary edema, potentially detrimental to the diffusion of oxygen into the blood and negatively affecting oxygen delivery. However, as seen in Fig. 3 and Table 3, PaO_2_ levels were not significantly different between the two groups, suggesting that the volume of fluid administered in this study positively impacted the cardiac output generated with CPR without negatively affecting oxygen diffusion.

Prior studies examining the effect of IVF during CPR have yet to answer the questions addressed in this study directly. Harris et al. [27] demonstrated that administering IVF during CPR increased carotid blood flow and arterial pressure generated by external chest compressions. Ditchey et al. reported that coronary blood flow during CPR is a function of the pressure difference generated across the arterial and venous vessels of the coronary system and occurs primarily during the relaxation (recoil) phase of each chest compression, with blood flowing passively into the coronary circulation at that point. The generation of a pressure differential during CPR necessary for blood to flow into the coronary circulation depends on a net transfer of blood from the venous to the arterial circulation [28]. Using the LUCAS 3 mechanical CPR device to deliver compressions allowed us to ensure that the compressions were delivered equally to all subjects in this study. This enabled any changes in the described pressure differential to be attributed to intravascular volume rather than variability in compression delivery within or across subjects. In hypovolemia, when both venous and arterial beds are highly compliant, CPR results in a small increase in transmural arterial pressure and a slight decrease in transmural venous pressure. Since vessel compliance decreases as a function of volume, a greater intravascular volume may be needed to create a more significant coronary arterial-to-venous pressure differential, thereby promoting greater coronary blood flow [22]. The impact of IVF on CO could be objectively measured by the COstatus—a device commonly used in many pediatric intensive care units for cardiovascular monitoring. This study technique is easily replicable in children who have suffered a cardiac arrest.

There are a few limitations in this study. For ethical reasons, it is challenging to perform CPR studies on humans, so most CPR studies are performed on animal or cadaver models. Inherent to any animal study is whether the results can be extrapolated to humans. The porcine model is often used due to the similarity in its physiology to human physiology and is a well-accepted model to study cardiac arrest physiology. A human study to answer the questions addressed in this study would not be possible, as the sophisticated hemodynamic monitoring performed in this study would not be possible in a cardiac arrest situation. Secondly, with the more convex shape of the thoracic cavity in pigs compared with humans, the compressions on a porcine CPR model may be suboptimal. However, we felt confident that high-quality CPR was maintained by ensuring that the end-tidal carbon dioxide measurement was consistently above 10 mmHg throughout the study. Thirdly, the volume of IVF administered during CPR may not have been the optimal volume for this purpose, and using more or less IVF could have resulted in better outcomes. The volume of IVF used in this study of 20 mL/kg was chosen because this is a widely used volume of IVF in resuscitative efforts and is generally adequate to improve stroke volume. Fourth of all, this CPR model does not precisely mimic how a cardiac arrest is managed, being that epinephrine was not administered. However, for this study, all factors were kept the same except for the intervention being evaluated; specifically, the use of IVF during CPR and its effect on CO. Administration of epinephrine during the CPR could have altered the CO, thereby confounding the results of this study, whether any change in CO was due to the IVF or due to the administration of an epinephrine dose. In addition, because the subjects were juvenile pigs, a ventilation rate of one breath every 3 s was used, consistent with the current Pediatric Advance Life Support guidelines [29]. Furthermore, there were some baseline differences between the two groups; most importantly, the CO was higher in the control group compared with the IVF group. The range we measured for baseline cardiac output measurements was consistent with prior studies on similarly sized and aged pigs. In a previous study performed in our laboratory, the baseline range of cardiac output in pigs of similar size and age was 1.4 to 5.2 L/min [8]. We attribute the difference in the baseline CO to an imbalance in the randomization resulting in more of the pigs at the higher end of the CO range being in the no-IVF group compared with the IVF group. However, we are confident in the results of this study since appropriate adjustments were made for differences in baseline variables. In addition, from the onset of the IVF administration, all the CO measurements in the IVF group were greater than those in the no-IVF group, resulting in a statistically significant augmentation in CO in the IVF group compared with the control group, from the effect of the IVF. Had there not been a baseline difference in CO between the two groups favoring the control group, we expect that the difference in CO between the IVF group and the control group seen during and after the IVF administration would likely have been even more significant. Lastly, it is unknown whether the improvement in cardiac output seen in this study will translate into improved patient-centered outcomes, including survival to hospital discharge and survival to hospital discharge with favorable neurological outcomes.

## Conclusions

This study demonstrated that the effect of IVF on CO and systemic oxygenation during CPR resulted in an increased CO after the initiation of IVF administration with statistically significant higher CO at 30-, 40-, and 50-min time points. In addition, systemic arterial oxygenation (PaO_2_) did not decrease as a result of the administration of IVF at any time point and remained above 100 mmHg in both groups throughout the study. Further studies should be conducted to determine if these outcomes translate into improved patient-centered outcomes in cardiac arrest patients and the optimal amount of fluid to administer during CPR.

## Data Availability

The datasets used and/or analyzed during the current study are available from the corresponding author upon reasonable request.

## Abbreviations

CPR: Cardiopulmonary resuscitation
CO: Cardiac output
FiO_2_: Fraction of inspired oxygen
IVF: Intravenous fluid
MSFP: Mean systolic filling pressure
OHCA: Out-of-hospital cardiac arrest
PaO_2_: Partial pressure of oxygen
PEEP: Positive end-expiratory pressure
Pra: Right atrial pressure
